# Some Practical Thoughts upon the Development of the Human Race and Obstetric Nursing*The author’s illness has prevented him from resuming these papers earlier.

**Published:** 1897-08

**Authors:** R. R. Kime

**Affiliations:** Atlanta, Ga.


### SOME PRACTICAL THOUGHTS UPON THE DEVELOPMENT OF THE HUMAN RACE AND OBSTETRIC NURSING.*

By R. R. KIME, M.D.,

Atlanta, Ga.

*The Room.*—This should be light, airy, well ventilated; if possible have sunlight and open fireplace. The water-closet should not open into it. The room should be clean, comfortably furnished, without extra drapery. If any case with contagious or infectious disease has previously occupied the room, it, with contents, should be properly disinfected. A floor oilcloth should be placed to the right side of the bed to protect carpet.

*The Bed.*—Spread a clean sheet over mattress, over this a rubber sheet. Pin over rubber a second sheet. On this spread a second rubber, then another sheet. The second rubber and sheet may be dispensed with if not convenient.

Under hips of patient two clean sheets folded, a clean quilt folded or a thick pad properly constructed may be used to catch the discharges. All should be clean, aseptic or sterilized—that is, boiled.

*Mother before Labor.*—She should have a general bath with soap and warm water just before labor; scrubbing inside of thighs and vulva well. If there is a redundancy of hair on vulva it should be trimmed off with scissors. She should be dressed in a short vest and nightgown. Over these a loose wrapper may be worn except in bed.

The patient should be placed on her back at right side of bed to facilitate examinations. Bladder and rectum should be emptied, using enema or catheter if they fail to act.

Patient may be allowed up and instructed *not* to "bear down" during pains in first stage of labor.

---

*Continued from April number. The author's illness has prevented him from resuming these papers earlier.

Nurse should not make any vaginal examinations nor alarm patient by telling of previous complicated cases.

*Second Stage of Labor.*—As a rule patient should be kept in bed with clothing folded and fastened well up under arms with a clean aseptic sheet pinned around body in the form of a skirt to catch discharges and protect clothing.

Unless second stage is slow patient should not be allowed to get out of bed even to evacuate bowels or bladder. Pulling upon hands of assistants or sheets tied to foot of bed aids expulsive pains, but should be avoided if labor is very rapid. Ergot should not be given in this stage of labor.

Chloroform may be used, if *properly given*, with but very little risk to patient in most cases. It should be administered under guidance of physician.

*Third Stage of Labor.*—The nurse may need to assist the physician by holding fundus of uterus, making slight pressure so as to make it contract down as child is expelled.

Patient's head should be immediately lowered if faint or much loss of blood; also promote uterine contractions by kneading uterus. Keep placenta for doctor's inspection. If child asphyxiated place in a pan of hot water—later in warm blanket—use artificial respiration if needed.

In absence of doctor prevent child being strangulated by secretions—when head is born if cord wrapped around neck slip it over head at once, hold head up, carrying upward as shoulders emerge from vulva, relieving weight and strain on perineum as much as possible.

Tie cord one inch or more from abdomen of child—then a second tying one inch or more from first; cut cord between, being careful not to injure baby with the scissors. Cleanse *eyes*, face and hands at once with a cloth wet in a disinfectant solution *to prevent ophthalmia*, then wrap in warm blanket.

Keep uterus contracted, especially if hemorrhage, by grasping fundus or kneading it until doctor arrives or nature expels placenta. Don't forget to lower head and raise foot of bed if hemorrhage.

*Mother after Labor.*—Shortly after labor, unless some *special*

contraindication exists, she should be cleansed, soiled clothing and bed-clothing removed.

When it can be properly carried out the antiseptic vulval pad and abdominal binder should be applied by or under the supervision of the attending physician, as it will add to the *comfort* and *safety* of the patient.

*At no time should the nurse handle the vulval dressings, remove or change the same, nor cleanse the patient without first washing and disinfecting her hands.*

The mother should keep her hands away from vulva during and after labor unless they have been properly cleansed. Except the first few hours after labor or in weak, debilitated patients, the mother should *be lifted up over vessel in bed for bowels to move or kidneys to act,* as such position favors drainage and passage of blood-clots. Any of the dressings, clothing or bedding that become soiled should be changed as soon as possible to prevent decomposition and infection of patient.

The discharges should be cleansed off three times in twenty-four hours, with a disinfectant solution, by *clean, disinfected hands.* The parts should be cleansed with disinfectant solution after each stool. Disinfectant solutions should consist of mercuric chloride, one in 2,000, three to five per cent. solution of creoline or carbolic acid, else boric acid, one tablespoon to one quart of warm water.

Mercuric chloride is best kept in tablet form, 7:33 gr., then one tablet to one, two, three and four pints of water make one in one, two, three and four thousand each. It is an active poison, should be handled with care, used in a wash-bowl or earthen vessel, as it corrodes most metals.

*Child.*—As soon as born should be wrapped in warm flannel and kept in a warm room. Eyes, face and hands should be immediately cleansed, preferably with a boric acid solution or creoline solution. Rub the skin with sweet oil, vaseline or lard, to facilitate removal of cheesy deposit. If very cold or child weak from any cause it should not be washed for several hours after birth, but simply oiled and rubbed off with a soft cloth.

Best to bathe baby of mornings, midway between nursing, using castile soap in warm water of about 90 degrees F. Avoid chilling surface, bathing not over five to ten minutes, drying quickly with a soft towel without friction, as skin is easily chafed.

The cord may be dressed with absorbent cotton, linen or aseptic gauze, dusting it with boric acid and placing to left side; over this a loose flannel belly-binder. The cord should be kept clean, dressings changed when soiled. If any pus or odor the physician should be notified.

When cord falls off or is removed, if there is any raw surface it should be cleansed off with boric acid solution and dressed with dry boric acid or borated vaseline. Napkins should be changed as soon as soiled, kept clean and dry. If folds of the skin become excoriated and inflamed, cleanse with weak solution of boric acid and dust with bismuth sub. nit. or chemically pure boric acid combined with equal parts of either oxide zinc or lycopodium.

Do not stuff baby with fat meat, teas and whisky toddies, as they tend to produce disease and do far more harm than good.

Unless some special condition prevents, allow baby to nurse soon after birth, as it stimulates uterine contractions in the mother and the infant obtains a secretion from breasts (colostrum) which acts as a laxative in moving its bowels.

At first baby should nurse every four hours until secretion of milk is established, then two to three hours, gradually lengthening the time to four hours by time it is three months old, not allowing it to nurse oftener than four to six hours of nights at any time.

Regular nursing at proper intervals during the day and at longer intervals of night will favor good digestion and prevent "colic."

Nothing is more harmful than nursing baby every time it cries, or stuffing it with teas or toddies; it adds fuel to the fire, increases the colic, disturbs the digestion and produces bowel troubles. Nurse the baby by the clock, allowing it a proper amount and sufficient time for one nursing to digest before taxing it with more work. The stomach needs a little rest, or at least sufficient time to accomplish the process of digestion. Many a mother, because the baby cries, not from hunger but from pain due to an overfed stomach, increases the trouble by giving it more milk, thus filling its "stomach" with two or three lots of milk in different processes of digestion, and wonders that the baby has the colic, bowel trouble and possibly dies. Then, to shift the responsibility, it is claimed that "God, in his kind providence, removed the child," when He had nothing to do with it, for it was either ignorance or wilful violation of physical laws.

Another thought worthy of careful consideration is the effect of the habit of dosing babies with whisky, soothing syrup and opiates, bringing the whisky bottle into service for every little ache or pain, thus laying the foundation for disease and appetites later in life.

If the mother's milk fails to agree with child the intervals of nursing should be lengthened, the amount lessened, and breasts kept empty by use of breast pump and the child fed some artificially prepared foods that require but little if any digestion, such as Liquid Beef Peptonoids, Panopeptones or Trophonine, one-third to one-half teaspoon of either, in water, four hours, half way between each nursing. Bowels should be kept moving each day with oil. The baby should be given tepid or warm water to drink between taking nourishment, and especially in place of letting it nurse. If mother's milk be scanty or wholly wanting, substitute sterilized cow's milk, malted milk, peptonized milk, etc.

Cow's milk is the best substitute for mother's milk, when it is properly sterilized and diluted with sterilized water and *slightly* sweetened with milk sugar or pure white sugar.

If milk fails to agree, barley water or *strained oatmeal water, well cooked*, may be combined with the milk to great advantage in many cases, especially after baby is a few months old and begins to acquire the power of starch digestion.

The proportion of water, sugar, cream, or starches to be added should be prescribed by the physician in each individual case.

---

Doctor—"You have only a few minutes to live. Have you any last wish?"

Patient—"Yes, I wish I had engaged another doctor."—*New York Tribune.*

### HOW TO PREPARE A FRACTURED FEMUR, OR OTHER BONE, FOR TRANSPORTATION.*

By C. H. RICHARDSON, M.D.,
MONTEZUMA, GA.

The treatment of railway injuries, on account of their environments, extent of injury to the soft parts, and very frequently the distance from the place of accident to the patient's home, must necessarily be somewhat different from cases that arise daily to the general surgeon; not like cases that we can see every day, or every hour, if need be, and by inspection see whether the parts are all right; that the circulation of the limb is good; but more often is it the case that we have to transport them long distances, that not only requires hours, but maybe days, before he arrives at his home or his journey's end. When this is the case, humanity not only demands such remedies as will relieve pain, but a splint, or dressing, that will keep the parts in perfect apposition, at the same time causing no danger from constriction.

In civil practice it is our custom to put the limb that is fractured at once in a plaster of Paris bandage; we are all united on this point, and we can get our patients in a few days on their crutches, if it is a fracture of the lower extremity; but in railway surgery, where the soft parts are generally contused and the patient has to travel a long distance to his home, the treatment by the plaster of Paris bandage at once is not safe; we cannot always tell what may be the condition of the limb twenty-four to forty-eight hours after the accident; therefore a constricting bandage may bring on not only great suffering to the patient, but might also produce mortification of the limb; then it is our duty to place the limb in a temporary splint that will hold the parts immovable, that will give him ease and comfort, and when he arrives at his journey's end he will be in as good condition as when we started him off. As I said before, the plaster of Paris bandage, if everything went well, would be preferable, but we are not sure that everything will go

*Read at meeting of Association of Railway Surgeons, Macon, Ga., April 20, 1897.

along well; the condition of the limb to-day is not positive indication of what will be the condition to-morrow; we can't see the patient while on his journey; we are not in a position to examine the limb, as to parts, and as to circulation, etc.

A temporary dressing can be made that will be perfectly comfortable to the patient, with no risk of gangrene and at the same time hold the parts immovable. I refer to the "gum shellac" splint, or Paris splint, open its entire length. The "gum shellac" splint is made by dissolving one pound of best gum shellac in one pint of alcohol and adding one dram of borax. To make the splint, cut out a piece of old woolen cloth long enough to reach from the extremity to the joint above the seat of fracture (I refer more especially here to the femur.) Then paint over one side of this woolen cloth this solution with a brush, and dry it thoroughly before the fire. When dry, add a second coat, which is also allowed to dry. Take one or more pieces of the same material and treat in the same manner; then when placed with their prepared surfaces together, which can be united by pressing with a hot iron, will not only make a strong splint when molded to the limb, but one that is flexible and porous. These splints can be prepared at any time, in our office, and when necessary to use them all we have to do is to trim them to suit the limb and gently heat them so as to mould them to the limb. With such a splint we first envelop the limb, in dressing a fractured femur, in cotton wadding; then cut the gum shellac splint so as to encircle the limb with the exception of one or two inches, so as to allow for inspection, gently heat it and secure it by a bandage, which can be allowed to remain on until the splint is thoroughly molded to the limb; then it can be removed. Here we have the limb lying in a well-padded cushion and kept in apposition by the splint.

The plaster of Paris splint, as recommended by Dr. Elliott, has proven highly successful; these can be made by taking two or three pieces of canton flannel and soaking them thoroughly in plaster of Paris, with an equal quantity of water; cut the pieces so as not to encircle the limb by one or two inches, and reaching from the extremity to joint above the seat of fracture; envelop the limb in cotton wadding as in the other splint, adjust the fracture and then apply the bandage so as to hold the parts in apposition until dry, then remove the bandage. This probably makes a stronger and a firmer splint than the gum shellac. In either of these splints, the gum shellac or plaster of Paris, the limb being left open its entire length to allow for inspection and prevent constriction, we have a splint that will keep the parts immovable, the limb easy and comfortable to the patient until he arrives at home or his journey's end; then either of the splints can be converted into a permanent one after his arrival at the end of his journey by applying a Paris bandage around it. If the fracture be compound, openings can be made into the splint so that antiseptic dressing can be applied.

---

With the July issue the *New Orleans Medical and Surgical Journal* celebrates its fiftieth anniversary. Excepting the years of the civil war it has been in active existence since 1844. In that year two young doctors (E. D. Fenner and A. Hester), finding themselves in New Orleans, their "fortunes alike desperate, without money, few acquaintances and dependent upon a precarious practice, determined to project the hazardous adventure of a Southern medical journal, and trust to the liberality of the profession for its support." The prospectus was printed on credit and the bill, eleven dollars, was paid with the first money which could be spared out of their combined wealth. A "poor French printer with a handful of type and nothing to do" was induced, "by means of flattering promises, to bring out the first number." Thus "the enterprise was conceived in poverty and poverty finally brought it forth." But each number managed to pay its own way, though, we are told, *without surplus*. From such beginnings the *New Orleans Medical and Surgical Journal* rapidly advanced to the front, where it has achieved an honorable name for itself and reflected credit upon Southern medical journalism. We congratulate our esteemed contemporary upon its past record; we are glad that it begins its fiftieth volume with such favorable indications of health and prosperity, and we trust that the melancholy days of *no surplus* have long since passed.

